# Vection lies in the brain of the beholder: EEG parameters as an objective measurement of vection

**DOI:** 10.3389/fpsyg.2015.01581

**Published:** 2015-10-13

**Authors:** Behrang Keshavarz, Jennifer L. Campos, Stefan Berti

**Affiliations:** ^1^Intelligent Design for Adaptation, Participation and Technology Centre for Rehabilitation Research, Toronto Rehabilitation Institute, University Health NetworkToronto, ON, Canada; ^2^Department of Psychology, University of TorontoToronto, ON, Canada; ^3^Department of Clinical Psychology and Neuropsychology, Institute for Psychology, Johannes Gutenberg University MainzMainz, Germany

**Keywords:** illusory self-motion, self-motion perception, objective measure, multisensory integration, event-related brain potentials (ERP), electroencephalogram (EEG), time-frequency analysis

## Current trends in vection research

Vection is typically defined as the subjective experience of self-motion in the absence of physical movement through space (Dichgans and Brandt, 1978; Hettinger et al., 2014). Vection is a common phenomenon in simulated environments such as driving/flight simulators, virtual reality (VR) interfaces, or video games (Riecke, 2011). Although vection was first described more than a century ago, the scientific interest in vection has recently been growing (see Frontiers in Psychology, 2015, *The Future of Vection* for a special issue devoted entirely to vection). Part of the motivation driving the need to better understand the mechanisms underlying vection comes from the fact that quantifying or characterizing vection may be critical to understanding its role in a variety of research approaches, theoretical assumptions, and applications. For example, outstanding questions include: (1) Is vection necessary to equate the behaviors elicited in simulated environments with those elicited in comparable real world environments? (2) Must a user experience true vection to optimize “transfer of training” effects across a variety of applications (e.g., transferability of driver training within a simulator to real world outcomes)?

If vection elicits perceptions and behaviors under certain circumstances that are comparable to those elicited during real physical movements through space, the application of vection-inducing techniques could have wide-ranging implications. The biggest challenge in addressing these questions is that there are no well-validated, objective measures that can reliably identify or characterize the experience of vection. Instead, vection is typically measured using non-standardized subjective rating scales that differ widely from study to study. The need for *objective* measurements is important for several reasons. For instance, the absence of objective and standardized measurements dramatically hampers the ability to compare across studies and verify the reproducibility of results. Further, any conclusions drawn from vection research (theoretical or applied) are based on a series of assumptions all hinging on the interpretation of qualitative data. In a recent review paper, Palmisano et al. (2015) emphasized the need for objective measurements of vection. In this paper we highlight EEG as a promising technique.

## EEG-based approaches to objectively measuring vection

### Key advantages of using EEG to study vection

The multifaceted EEG signal enables the measurement of cortical activity on the surface of the scalp. It allows for temporally precise, online measures of the working brain. In other words, EEG offers access to the neuro-cognitive processes underlying vection. Compared to other imaging techniques [e.g., functional magnetic resonance imaging (fMRI)], EEG is portable (i.e., applicable in different experimental settings including immersive displays/simulators, allowing investigators to extensively and systematically probe vection responses across different sensory conditions and contexts), inexpensive, and can be easily administered. In addition, the acquisition of the EEG is only of minimal burden to participants and is usually well tolerated. Below we summarize some of the main advantages of applying EEG to vection research:

- *EEG does not require an overt response from the participant*. Vection onset and strength are typically measured using subjective responses (button press, verbal response), however, EEG methods allow investigators to compare neural responses under different conditions without requiring an active behavioral response. Consequently, vection can be measured in situations when subjective responses are impractical or impossible. They can also be used in conjunction with subjective responses as a method of cross-correlating consciously reported experiences with associated neural responding.
- *EEG has a high temporal resolution*. EEG signals provide information with millisecond resolution far exceeding the temporal resolution of other imaging techniques such as fMRI or positron emission tomography. This does not only enable tapping into the dynamics of neuro-cognitive processing that are characterized by continuous changes in brain activity, but also allows one to focus on different points within the time course of responding to the vection inducing stimulus (e.g., vection-onset/-offset).
- *The EEG signal is well-established*. A long history of application of EEG in basic research has resulted in different analysis techniques. Due to the widespread use of EEG, factors that may impair the quality of the signal (e.g., eye-movements, head-movements) have been well documented and accounted for in developing experimental protocols and when performing data reduction and analyses. This allows capturing physiological correlates of vection in settings that do not guarantee high data quality, for instance, due to body movements or interfering signals. Other potential physiological measurements do not offer a comparable range of data processing techniques.
- *The EEG signal is multifaceted and extensive*. The EEG signal contains a great deal of information about the dynamics of the brain and, as such, enables extracting unique vection-related parameters. Different EEG measures such as event-related brain potentials (ERP) and time-frequency analyses have already been introduced in the context of vection research, but additional options are feasible. For instance, a novel analytical tool is the so-called coherence analysis, which is applied to EEG data in order to identify functional (local or global) neural networks that may contribute to the sensation of vection.

### Empirical research to date using EEG to study vection

In addition to the theoretical advantages of using EEG, there is a limited but growing body of empirical research that has used EEG during vection, providing evidence of its applicability and potential impact. For instance, Thilo et al. (2003) used EEG to compare vection-inducing stimuli with stimuli perceived as object-motion and demonstrated significant differences in early cortical activity (N70) when vection was experienced. More recently, Keshavarz and Berti (2014) measured ERPs during an initial brief period of stimulus presentation (approx. 2 s) for stimuli that induced vection when they were shown for a longer duration (45 s). Results demonstrated that the stimulus that generated the strongest vection when presented for 45 s (measured by subjective ratings) also elicited pronounced ERPs at the very early stages of cognitive processing that occurred during the initial, brief presentation (P1, N2). Consequently, the authors hypothesize that a vection-specific neural pattern may be observed in the EEG signal even before the subjective sensation of vection is reported. However, note that this conclusion must be confirmed by future studies that measure cortical activity during (and not only before) actual vection.

The temporal resolution of EEG may also provide unique insights into the different stages of vection and may allow for a better approximation of the timescale of these events. While a comprehensive study characterizing changes across the various timescales has not yet been conducted, evidence across studies suggest that there may be differences in the EEG signal associated with vection onset, length, and strength. As previously mentioned, Keshavarz and Berti (2014) examined ERP differences during the initial processing of briefly presented stimuli that could generate vection when presented for a longer time (i.e., presumably pre-vection). A study by Barry et al. (2014) focused on EEG correlates during vection and showed desynchronization in the low alpha and gamma band. Thus, it not only seems possible to *identify* the onset of the perception of vection during sensory stimulation (Wiest et al., 2001; Barry et al., 2014), but also to potentially *predict* the perception of vection at an initial state of stimulus processing.

Importantly, there is also now emerging research describing EEG signals that occur in the presence of actual self-motion through space (see Nolan et al., 2009, for passive self-motion and Gramann et al., 2014, for active locomotion). Novel techniques have now been developed to account for and remove artifacts created by the physical movement itself. As such, these studies provide a juxtaposition to evaluate how the EEG signals elicited during vection-inducing stimulation compare to the EEG signals elicited during actual self-motion.

### Potential areas of particular impact resulting from EEG-vection studies

In general, the experience of vection has been described as a potentially important phenomenon for VR-based applications (Riecke, 2011; Hettinger et al., 2014; Riecke et al., 2015). There are tremendous opportunities to exploit the data obtained through EEG-vection studies to advance numerous theoretical and applied areas of research. For example, a continuous, objective measure correlated with subjectively reported vection would be particularly impactful in the context of VR applications necessitating simulated self-motion such as driving or flight simulation. If a “signature” EEG pattern associated with vection was discovered, this could be used to compare simulated movements with real movements to determine how they are similar/different with respect to this signature pattern. Understanding these differences might help to target ways to optimize simulations in order to, for instance, enhance the transfer of knowledge and/or training derived in the simulator to the intended real world application.

Furthermore, an objective EEG-based measurement of vection might be important in the context of physical rehabilitation applications. For instance, effective stroke rehabilitation depends on accurate perceptual-motor coupling that enables appropriate adaptation and relearning of sensorimotor associations. Simulator or VR systems that induce vection may offer enriched rehabilitation techniques that may arguably foster more precise perceptual-motor coupling and therefore deliver better and more rapid functional outcomes. In addition, such approaches may reduce the gap between simulated and real life conditions and, as such, may allow for more effective transfer effects from rehabilitative interventions to everyday life.

## Challenges in validating EEG as an objective measure of vection

Using EEG to objectively measure vection is not trivial and several challenges exist. Most importantly, the validation of an objective measurement tool that is used to measure a subjective experience must, by nature, be associated with a subjective response (at least initially). In order to address this concern, it is important to cross-compare data from converging sources and across different objective and subjective measurements using the same paradigm to account for differences across unique approaches. Furthermore, additional novel tasks must be considered to complement both the traditional approaches used to measure vection as well as the proposed objective measures such as EEG. As one example, it is known that *imagining* self-movements through space often leads to different spatial updating than *actual physical movements* (Klatzky et al., 1998; Campos et al., 2009). Therefore, implementing these types of established spatial updating paradigms under conditions in which vection is present or absent may provide a unique assay of whether vection more closely approximates *real* vs. *imagined* self-motion (and importantly how these intuitive behavioral responses are then associated with objective EEG responses).

Another challenge associated with EEG measures is that the signal can reflect parameters that are not purely vection-related, including specific types of visual stimulation (e.g., global visual motion) or attentional demands not related to self-motion. Consequently, it is important to separate brain activity that solely stems from sensory processing from brain activity that reflects the neuro-cognitive processes associated with sensory integration (presumably underlying vection; Keshavarz and Berti, 2014).

Overall, extracting an EEG signature that precisely represents neural responses related exclusively to vection will require intensive investigation. The first step should be to implement several existing techniques that allow for the specific extraction of relevant information from the EEG signal and that can reduce the influence of artifacts associated with motion (e.g., independent component analysis; Delorme and Makeig, 2004). Subsequently, systematic comparisons of EEG-correlates of vection with other potential vection parameters (eye-movements or postural responses, Palmisano et al., 2015) and with subjective ratings are mandatory in order to validate EEG-based vection measurements.

## Conclusion

In our opinion, the future of vection research relies on the development and application of objective measures to complement traditional approaches. We have argued that a promising candidate for this type of measure includes parameters derived from EEG. This conclusion stems from the strong potential of the multifaceted EEG signal to deliver a variety of different metrics. Recent studies have already determined some parameters of potential interest, however, these studies are only the starting point for EEG-based vection measures. We believe that future research will identify various EEG parameters that may help to elucidate the nature and the neuro-cognitive basis of vection. These parameters could be used to re-evaluate previous theoretical assumptions and empirical findings in the context of vection research, provide guidance in the development of novel experimental paradigms and protocols, and could help to direct future work within applications for which vection might be particularly important.

## Funding

This paper was partially supported by a grant received from the German Research Foundation (DFG; BE2678/3-1).

### Conflict of interest statement

The authors declare that the research was conducted in the absence of any commercial or financial relationships that could be construed as a potential conflict of interest.
